# Relationship between Fatigue and Physical Activity in a Polish Cohort of Multiple Sclerosis Patients

**DOI:** 10.3390/medicina56120726

**Published:** 2020-12-21

**Authors:** Michalina Rzepka, Mateusz Toś, Michał Boroń, Katarzyna Gibas, Ewa Krzystanek

**Affiliations:** 1 Department of Neurology, Faculty of Medical Sciences in Katowice, Medical University of Silesia, 40-752 Katowice, Poland; michalina.rzepka93@gmail.com (M.R.); mateusz.tos@sum.edu.pl (M.T.); kgibas@sum.edu.pl (K.G.); 2 Department of Gynecology and Obstetrics, Faculty of Medical Sciences in Katowice, Medical University of Silesia, 40-752 Katowice, Poland; michal.boron123@gmail.com

**Keywords:** multiple sclerosis, fatigue, physical activity, disability, professional activity

## Abstract

*Background and objectives:* Fatigue is one of the most common and disabling symptoms of multiple sclerosis (MS). It can be defined as a subjective lack of physical and mental energy. The aim of this study was to evaluate the frequency and severity of fatigue in patients with MS and its relationship with overall physical activity and disease-related disability. *Materials and Methods:* The study included 100 patients with a clinical relapsing-remitting form of MS. Patients with severe depression were excluded. Neurological impairment was rated using the Expanded Disability Status Scale (EDSS). Fatigue was assessed using the Modified Fatigue Impact Scale (MFIS) and the Fatigue Severity Scale (FSS), with FSS scores greater than 36 indicating patients with fatigue. Physical activity was evaluated with the International Physical Activity Questionnaire (IPAQ) and categorized on three levels: low, moderate, and high, using standard metabolic equivalents (MET). *Results:* The average FSS and MFIS scores were (mean ± SD) 31.3 ± 15.2 and 30.1 ± 17.0, respectively. The mean EDSS score was 2.5 ± 1.5. 42%. Patients were classified as fatigued based on FSS. Fatigued patients had higher mean EDSS scores than non-fatigued (3.0 ± 1.6 vs. 2.2 ± 1.4, respectively, *p* = 0.002). Low, moderate, and high levels of physical activity were reported in 35%, 20%, and 45% of patients, respectively. Higher scores of fatigue in FSS and MFIS were inversely correlated with the intensity of physical activity (r = −0.38, *p* < 0.001 and r = −0.33, *p* < 0.001, respectively). *Conclusions:* In patients with MS, fatigue is a common symptom. Patients with lower physical activity and greater MS-related disability have a higher severity of fatigue, which negatively affects cognitive, psychosocial, and physical functioning.

## 1. Introduction

Multiple sclerosis (MS) is a chronic, inflammatory-demyelinating disease of the central nervous system [1]. There are over 2 million people suffering from MS worldwide and approximately 40,000–60,000 in Poland [2,3]. Moreover, it is one of the main causes of disability among young people [4,5]. Multifocal cerebral damage is characterized by a large variety of symptoms, such as paresis, sensation deficits, or balance disorders, which are often accompanied by mental and cognitive disorders or excessive fatigue [6].

Fatigue occurs in many pathological conditions, whereas MS belongs to neurological diseases most often accompanied by fatigue. It is defined as a subjective feeling of a lack of energy to start and sustain any activity, which is unrelated to depression or muscle weakness. In contrast to tiredness, fatigue is characterized by longer duration, lack of improvement after rest, intensification under the influence of heat, and the peak of intensity occurring in the morning. Until now, no clear cause of fatigue has been found. It is believed that pathophysiology is complex and multifactorial. In many neurological disorders, fatigue is perceived as one of the most disabling symptoms, which has a strong impact on every aspect of the patients’ life, e.g., quality of life or social and professional functioning [7,8,9,10,11,12,13]. For clinicians, it is important to understand the relationship of fatigue with the progress of MS and its association with patients’ physical performance.

Therefore, the objective of this study was to assess the prevalence and severity of fatigue among patients with MS and its relationship with overall physical activity and disease-related disability.

## 2. Materials and Methods

The study involved 100 Polish patients with a relapsing-remitting form of MS (RRMS), treated in the Neurological Outpatient Clinic and Department of Neurology of our institution from December 2016 to March 2018. All participants had to have confirmed diagnosis of RRMS, according to the 2010 Revised McDonald criteria [14]. The exclusion criterion was severe depression, determined with Beck’s Depression Inventory (BDI). The study was performed in compliance with the Declaration of Helsinki. Ethics committee approval was not required as this was a questionnaire-based survey, which did not fulfil the criteria of a medical experiment. All participants gave their oral informed consent before providing any information to the questionnaire.

In the first part of the questionnaire, we collected demographic and socioeconomic data (gender, age, educational level, marital status, place of residence, smoking, profession), as well as basic clinical information (comorbidities, duration of MS, first symptoms, course of the disease, present and previous treatment, and number of relapses).

Fatigue was assessed using two scales: The Fatigue Severity Scale (FSS) [15] and the Modified Fatigue Impact Scale (MFIS) [16]. The International Physical Activity Questionnaire (IPAQ) [17] was used to assess physical activity and BDI was used to evaluate depressive disorders [18,19].

The FSS is the most common tool used to assess severity of fatigue, through nine items concerning various aspects of fatigue over the last week. Higher scoring indicates greater severity of fatigue, with a maximum score of 63. A score above 36 is considered abnormal and indicates fatigue [8,9,10,15,20]. In this study, we have implemented this cut-off to identify participants with fatigue and to assess the overall incidence of fatigue in patients with MS.

The MFIS consists of three subscales, which assess impact of fatigue on physical (F-1, 9 items), cognitive (F-2; 10 items), and psychosocial functioning (F-3; 2 items) over the last four weeks [16]. The respondent answers the question by determining the frequency of specific disorders. The result ranges from 21 to 105 points. The higher the value of the result, the greater the effect of fatigue on the patient’s functioning [7,16,21,22,23].

The Polish version of the IPAQ short-form consists of seven questions to indirectly evaluate physical activity throughout the last week prior to the survey [18,24]. Questions concern physical activity related to everyday life, work, and leisure. Physical activity was expressed as a standard metabolic equivalent (MET) of the task (unit of work/kg body weight/hour) in MET-min/week [25]. Depending on the result, patients were categorized to three groups of different levels of physical activity: low (below 600 MET-min/week), moderate (600–1499 MET-min/week), or high (at least 1500 MET-min/week) [18,24].

BDI was used to evaluate possible exclusion criteria: the presence and severity of depressive symptoms. It was a self-administered questionnaire containing 21 questions covering a period of the past 2 weeks. Higher scoring indicated greater severity of depression with the maximum score of 63, and scores of 30 or greater indicate severe depression [18,19,26].

To quantify disability and neurological impairment, the Expanded Disability Status Scale (EDSS) was used [27]. The EDSS scores range from 0 (no disability) to 10 (death due to MS) in 0.5-unit increments. Higher scores indicate greater disability, with scores from 1.0 to 4.5 assigned to patients who are able to walk without any aid and scores from 5.0 to 9.5 assigned to patients with different levels of walking impairment. This assessment was performed by an experienced neurologist, who was certified in administering the scale.

Statistical analysis was performed with the Statistica 13.0 software (TIBCO Software Inc., Palo Alto, CA, USA) [28]. For assessing distribution, we used the Shapiro-Wilk test. The between-group comparisons were performed with a *t*-test, chi-squared test, Mann-Whitney U test, or ANOVA, with post-hoc pairwise comparisons performed using Dunn’s multiple comparison test. Correlations were assessed using the Pearson correlation coefficients or the Spearman correlation coefficients. Significance was determined at the level of *p* < 0.05.

## 3. Results

### 3.1. Fatigue—General Results

The study group consisted of 100 patients with RRMS; 78 women and 22 men, aged 38.8 ± 9.8 years (mean ± SD), with an age range of 19–65 years. The average FSS score was 31.3 ± 15.2 points, and 42 patients (42%) had scores greater than 36. Patients with fatigue had a higher mean score of EDSS than patients without fatigue (3.0 ± 1.6 vs. 2.2 ± 1.4, respectively, *p* = 0.002) and a longer mean duration of the disease than those without fatigue (9.6 ± 5.2 years vs. 7.1 ± 6.9, respectively, *p* = 0.003). Demographic, socioeconomic, and clinical data of the studied group are summarized in Table 1.

### 3.2. Fatigue and Physical Activity

**Figure 1:**
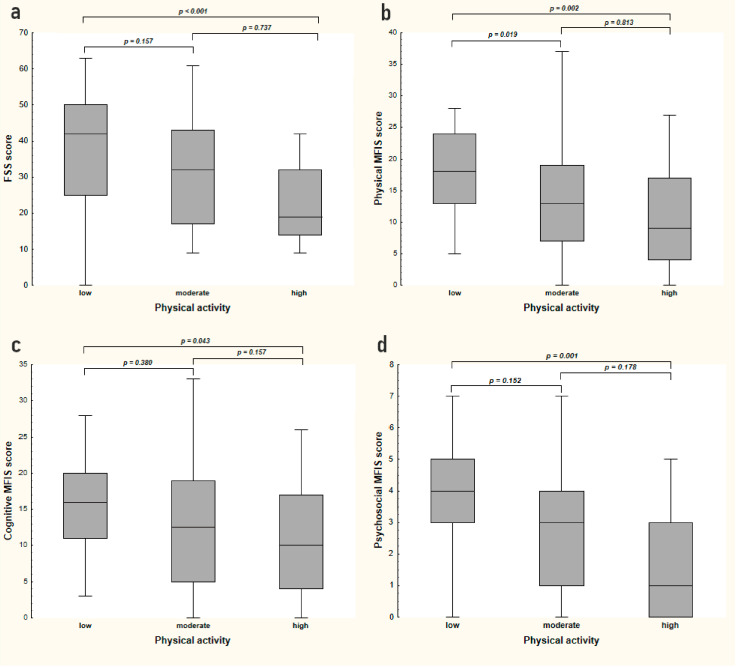
Intensity of fatigue in three groups of patients with MS categorized by their level of physical activity: low (*n* = 35), moderate (*n* = 20), and high (*n* = 45). Engagement in physical activities (low, moderate, and high) assessed with the International Physical Activity Questionnaire (IPAQ). Intensity of fatigue assessed with the Fatigue Severity Scale (FSS) (**a**) and the Modified Fatigue Impact Scale (MFIS) (**b**–**d**). Higher scores in fatigue scales represent greater fatigue perceived. Between-group comparisons were performed with ANOVA (*p* < 0.05 in each scale) and presented pairwise comparisons performed with the post-hoc Dunn’s multiple comparison test. The central line of the box represents the median score; the top and bottom of the box represent the 1st and 3rd quartiles; and the ends of the whiskers represent the minimum and maximum values.

According to the IPAQ, 35% of subjects had low (below 600 MET-min/week), 20% moderate (600–1499 MET-min/week), and 45% high (above 1500 MET-min/week) levels of physical activity over the previous 7 days. The average level of physical activity measured with MET was significantly lower in patients with fatigue than in those without (mean ± SD; 1294 ± 2317 vs. 2860 ± 3038, respectively, *p* < 0.001) (Table 1), and overall FSS scores were inversely correlated with levels of physical activity (r = −0.38, *p <* 0.001) (Table 2). Similar results were found in the total MFIS score (r = −0.33, *p* < 0.001) and in physical and psychosocial subscales of the MFIS (r = −0.36, and r = −0.36, *p* < 0.001, respectively). Subjects who were more physically active also had lower levels of fatigue in the MFIS cognitive score (r = −0.25, *p* = 0.012) (Table 2 and Figure 1).

### 3.3. Fatigue and Disability

Mean EDSS scores were higher in patients with fatigue than in those without (3.0 ± 1.6 vs. 2.2 ± 1.4, respectively, *p* = 0.002) (Table 1). Greater neurological disability assessed with EDSS was associated with higher levels of fatigue, measured with FSS, and higher impact of fatigue on patients’ functioning, measured with all MFIS scores (Table 2).

### 3.4. Fatigue and Disease Duration

Patients with fatigue had MS longer than those without (9.6 ± 5.2 vs. 7.1 ± 6.9 years, respectively, *p* = 0.003) (Table 1). Duration of MS was positively correlated with the impact of fatigue on a patient’s life, measured with MFIS total physical, psychosocial, and cognitive scores (Table 2).

### 3.5. Fatigue and Professional Status

Among professionally active patients (overall 62%), a higher proportion of participants was categorized as non-fatigued based on the FSS score than those not professionally active (68% vs. 47%, respectively, *p* = 0.044). Moreover, professionally active patients had overall lower MFIS total scores than those who were non-active (27.5 ± 17.4 vs. 34.3 ± 15.7, respectively, *p* = 0.046).

### 3.6. Fatigue and Gender, Place of Residence, or Smoking

No significant correlations were found between severity or impact of fatigue and patients’ gender, place of residence, or cigarette smoking.

## 4. Discussion

Our study showed that the prevalence of fatigue among Polish patients with a relapsing-remitting form of MS, who were not substantially disabled or limited in their functioning (mean EDSS of 2.5 ± 1.5), is quite high (42%). According to Lobentanz et al., for 60.3% of patients with MS, fatigue is one of the three most troublesome symptoms of the disease, and for 64.1%, it significantly affects work and family life [29]. Many investigators proved that fatigue has a significant impact on quality of life [30,31,32,33]. Similar results to ours have been reported in other studies. Runia et al. found the occurrence of fatigue in 46.5% of patients with clinically isolated syndrome (CIS) [34]. Simpson et al. examined almost 300 patients, mostly with low levels of disability (median EDSS 1.5), detecting fatigue in 41.3% [35]. However, in many publications, the prevalence of fatigue is higher, on average between 55−80% [8,13,29,30,36]. The differences between the results may be due to the fact that the authors included MS patients with a progressive form of the disease, thus with a longer duration of the disease and a higher level of disability [8,13,29,30]. We should consider that both methods of fatigue testing (the FSS and the MFIS) have some limitations, which may be also the reason for some underestimation. The FSS only determines the severity of fatigue, whereas the MFIS measures the impact of fatigue on a patient’s life. Grading in MFIS subscales (physical, cognitive, and psychosocial fatigue) does not take into account a possible severity of deficits contributing to observed limitations [37].

The analysis showed that patients with higher neurological disability, according to the EDSS, were significantly more fatigued. We also found that a higher level of fatigue occurs in patients with a longer duration of the disease. Many other studies reported similar conclusions [29,38,39,40,41,42]. Interestingly, some studies confirmed correlation between fatigue and higher disability, but not with the disease duration [35]. This can be explained by the heterogeneous course of MS and the fact that the primary progressive form has a worse course than the relapsing-remitting form. The course of the disease may be mild and neurological disability may not correlate with the disease duration.

This study confirmed a negative correlation between the level of fatigue and intensity of physical activity. The more physically active the patient, the lower the level of fatigue. These results were found in the total MFIS score for all subscales and in FSS score, as well. According to the World Health Organization (WHO), physical activity is defined as any skeletal muscle activity exerted by an individual resulting in energy expenditure [43]. The recommended intensity of physical activity for people with chronic conditions refers to moderate aerobic activity (50–70% of maximum heart rate) of at least 150 min a week or vigorous aerobic activity (70–85% of maximum heart rate) of at least 75 min a week, or a combination of both each week [43]. It was challenging to provide a more detailed recommendation for exercise, since, in our research, intensity of physical activity was measured by a self-reported questionnaire. Over the years, many studies have confirmed that higher physical activity improves fatigue [20,35,44,45,46,47,48,49,50,51,52], although the pathophysiology of fatigue in MS is multifactorial and still not fully understood. Langeskov-Christensen’s study shows that exercise can alleviate fatigue on both the primary and secondary pathways [44]. It is still unknown which exercises have the most beneficial effect on fatigue. According to Latimer-Cheung et al., exercise of moderate intensity performed twice a week is effective in improving mobility and fatigue in MS patients with mild to moderate disability [53]. Moreover, it seems that resistance training with the use of weights or aerobic training may be most effective for reduction of fatigue [53]. The positive effect may be the result of aerobic capacity and muscle strength improvement, although there are studies demonstrating that aerobic training does not reduce fatigue [54]. It should be noted that, when recommending physical activity, exercises ought to be individually adjusted to the patient’s abilities. Inconsistency about the best type of physical activity between studies makes it difficult to draw meaningful recommendations for exact type of exercise or training [21,48,49,50,54]. 

Adequate physical activity has a positive impact on the health of patients with chronic diseases [55]. It has been proven that properly selected exercises improve the physical and mental condition in patients with chronic fatigue syndrome (CFS) and are often implemented together with the other forms of therapy [56,57,58,59,60,61]. However, some researches question the clinical efficacy of graded-exercise-related therapy (GET) [62], while other sources even report deterioration after GET [63,64]. Among several patients with myalgic encephalomyelitis/chronic fatigue syndrome (ME/CFS), the post-exertional “malaise” (PEM) was observed, which can be defined as a ‘pathological inability to produce sufficient energy on demand’. Excessive activity can lead to PEM, so it is essential to find a balance between rest and activity for PEM prevention [61,65,66]. In our study, we did not observe such a phenomenon, nor did we determine the optimum level of activity. Interestingly, Janson et al. compared ME/CFS with MS and reported significantly more functional limitation and more severe symptoms in ME/CSF patients than those with MS [67].

Interestingly, more physically active patients were also mostly professionally active. We showed a marked reduction in fatigue in the professionally active group compared to unemployed or pensioners. Similar conclusions are confirmed by other studies [8,13,35]. On the other hand, it should be noted that chronic fatigue is one of the main causes of unemployment among patients with MS [68,69,70].

Our results confirmed previous observations, that the incidence and level of fatigue is not different between genders in patients with MS [8,13,22,34,71,72,73]. However, some studies indicate that fatigue may be more frequent or more severe in women [7,35]. The explanation of gender differences requires further research.

This study has several limitations. It was based on a self-report questionnaire, which entails the risk of a reporting bias and some associated confounding factors. The study group might not be representative of the entire population of MS patients (e.g., a higher prevalence of women, only the relapsing-remitting course of the disease, average low disability). We also did not investigate the differences in the effect of physical activity on fatigue and the type of exercise. Taking into consideration the limitations of this study, further research is needed.

Fatigue, due to its high prevalence and significant impact on the quality of life, is a crucial problem among patients with MS. In clinical practice, we often observe insufficient effectiveness of pharmacological treatment. To effectively reduce the occurrence of fatigue, further research should be performed to understand its etiology. It is also important to identify other factors that can reduce fatigue. It seems that physical involvement associated with professional activities may represent protective measures for fatigue in MS patients or contribute to a decrease of its level, despite a long duration of illness. The specific impact of physical activity could contribute to the creation of new recommendations and thus to the improvement of a patient’s quality of life.

## 5. Conclusions

In patients with MS, fatigue is a common symptom. Patients with lower physical activity and greater MS-related disability have a higher severity of fatigue, which negatively affects cognitive, psychosocial, and physical functioning. Fatigue is more severe in MS patients with longer disease duration and higher levels of disability.

## Figures and Tables

**Table 1:** Demographic, socioeconomic, and clinical characteristics of the studied patients with relapsing-remitting multiple sclerosis (MS), categorized as fatigued and non-fatigued based on Fatigue Severity Scale (FSS) scores.

Characteristics	Whole Group (*n* = 100)	Fatigued (*n* = 42)	Non-Fatigued (*n* = 58)	*p*
Gender *n* (%)				
Women	78 (78%)	85.7%	72.4%	0.110 *
Men	22 (22%)	14.2%	27.6%	
Age (years) (mean ± SD)	38.8 ± 9.8	41.0 ± 9.4	37.3 ± 9.9	0.071 †
Marital status *n* (%)				
Single	40 (40%)	33.3%	44.8%	0.247 *
In relationship	60 (60%)	55.2%	44.8%	
Place of residence *n* (%)				
Rural areas	12 (12%)	14.3%	10.3%	
Town (population):				
up to 50,000	18 (18%)	14.3%	20.7%	0.616 *
50,000–99,999	18 (18%)	14.3%	20.7%	
100,000 and more	52 (52%)	57.1%	48.3%	
Smoking *n* (%)				
Yes	12 (12%)	4.8%	17.2%	0.058 *
No	88 (88%)	95.2%	82.8%	
Education *n* (%)				
Vocational	12 (12%)	19.0%	6.9%	
Secondary	32 (32%)	42.9%	24.1%	0.007 *
Higher	56 (56%)	38.1%	69.0%	
Duration of the disease (years) (mean ± SD)	8.19 ± 6.34	9.6 ± 5.2	7.1 ± 6.9	0.003 ‡
EDSS (mean ± SD)	2.5 ± 1.5	3.0 ± 1.6	2.2 ± 1.4	0.002 ‡
MET (mean ± SD)	2234 ± 2865	1294 ± 2317	2860 ± 3038	<0.001 ‡
MFIS total score (mean ± SD)	30.0 ± 17.0	42.4 ± 13.0	21.2 ± 13.7	<0.001 †
MFIS physical score (mean ± SD)	14.1 ± 8.4	19.9 ± 6.4	10.0 ± 7.1	<0.001 ‡
MFIS cognitive score (mean ± SD)	13.2 ± 8.0	18.6 ± 7.0	9.4 ± 6.3	<0.001 †
MFIS psychosocial score (mean ± SD)	2.8 ± 2.0	4.1 ± 1.4	1.8 ± 1.7	<0.001 ‡

Fatigued and non-fatigued patients were categorized based on FFS, where scores up to 36 indicated non-fatigue. Multiple sclerosis (MS); Fatigue Severity Scale (FSS); Metabolic equivalent (MET) estimated with International Physical Activity Questionnaire (IPAQ); Modified Fatigue Impact Scale (MFIS); Expanded Disability Status Scale (EDSS); standard deviation (SD); *p*—values of statistical significance for comparisons of fatigued and non-fatigued patients; *—chi-squared test; †—student’s *t*-test; ‡—Mann–Whitney U test.

**Table 2:** Correlations of demographic, socioeconomic, and clinical characteristics with fatigue scores in studied patients with relapsing-remitting MS.

Characteristics		FSS Score	MFIS Total Score	MFIS Physical Score	MFIS Psychosocial Score	MFIS Cognitive Score
Age	R	0.17 *	0.27 †	0.32 *	0.38 *	0.18 †
	*p*	0.09	0.00	0.00	0.00	0.09
Place of residence	R	0.00 *	0.02 *	−0.02 *	0.02 *	0.03 *
	*p*	0.97	0.84	0.88	0.86	0.76
Smoking	R	−0.04 *	−0.02 *	0.01 *	0.02 *	−0.11 *
	*p*	0.70	0.86	0.95	0.83	0.28
Education	R	−0.21 *	−0.23 *	−0.21 *	−0.28 *	−0.21 *
	*p*	0.04	0.02	0.03	0.01	0.03
Duration of the disease	R	0.30 *	0.27 *	0.37 *	0.37 *	0.29 *
	*p*	0.00	0.00	0.00	0.00	0.00
IPAQ (MET)	R	−0.38 *	−0.33 *	−0.36 *	−0.36 *	−0.25 *
	*p*	0.00	0.00	0.00	0.00	0.01
EDSS	R	0.36 *	0.45 *	0.49 *	0.54 *	0.29 *
	*p*	0.00	0.00	0.00	0.00	0.00

Fatigue Severity Scale (FSS); Modified Fatigue Impact Scale (MFIS); Metabolic equivalent (MET) estimated with International Physical Activity Questionnaire (IPAQ); Expanded Disability Status Scale (EDSS); *—Spearman’s rank correlation coefficient; †—Pearson correlation coefficient.
